# Generation of Primordial Germ Cell-like Cells from hESCs Using BMP4 and hAFSC-Conditioned Medium

**DOI:** 10.3390/mps9020035

**Published:** 2026-02-28

**Authors:** Borislav Arabadjiev, Ivelina Vassileva, Georgi Nikolaev, Roumen Pankov

**Affiliations:** Faculty of Biology, Sofia University St. Kliment Ohridski, 1164 Sofia, Bulgaria

**Keywords:** primordial germ cell-like cells (PGC-LCs), human embryonic stem cells, bone morphogenetic protein 4 (BMP4), human amniotic fluid stem cells (hAFSC), germline differentiation, conditioned medium

## Abstract

The differentiation of human embryonic stem cells (hESCs) into primordial germ cell-like cells (PGC-LCs) provides a robust in vitro model to study human germline specification. Here, we present a simple, reproducible, and cost-effective protocol for generating DEAD-box helicase 4 (DDX4)/VASA and Deleted in Azoospermia-Like (DAZL)-positive PGC-LCs from hESCs using a combination of bone morphogenetic protein 4 (BMP4) and conditioned medium (CM) derived from Stage-Specific Embryonic Antigen-4 (SSEA4)-positive human amniotic fluid stem cells (hAFSC-4). Importantly, unlike conventional protocols that rely on embryoid body formation, our method employs adherent cultures for germ cell differentiation. This approach enhances reproducibility by avoiding the spontaneous and stochastic variability inherent to embryoid body formation. This protocol provides a reproducible and physiologically relevant platform for studying human germ cell development in vitro.

## 1. Introduction

Developing robust protocols for differentiating human embryonic stem cells (hESCs) into primordial germ cells is of significant importance for both basic and translational research. Primordial germ cells are the precursors of gametes, and in vitro models provide a unique opportunity to investigate early germline specification and development—processes that are otherwise inaccessible in humans. Such protocols enable the study of infertility-related pathologies, facilitate the modeling of epigenetic reprogramming and imprinting disorders, and support the exploration of mechanisms underlying germline-associated diseases. Furthermore, establishing reproducible and developmentally faithful methods ensures rigor and safety in potential applications ranging from regenerative medicine to patient-specific fertility treatments. Importantly, the in vitro derivation of primordial germ cells also offers an ethically viable alternative to studying early human germline development in vivo.

Human germline development is orchestrated by a tightly regulated interplay between intrinsic transcriptional programs and extrinsic niche-derived cues. During early mouse embryogenesis, primordial germ cells (PGS) are specified in the epiblast under the influence of signals from extraembryonic mesoderm, most notably bone morphogenetic protein 4 (BMP4) [1]. For some time, this model was extrapolated to primates and humans as the presumed mechanism of germ cell specification. However, more recent data demonstrate that in cynomolgus monkeys, PGCs are specified in the dorsal amnion through BMP4 signaling, which activates downstream regulators such as SOX17, BLIMP1, and TFAP2C, thereby initiating the germ cell fate program [2,3]. Beyond BMP signaling, the embryonic niche provides a complex mixture of cytokines, growth factors, and extracellular matrix interactions that support PGC survival, proliferation, and maturation.

Recreating this microenvironment in vitro has proven challenging. Traditional differentiation approaches using hESCs have relied primarily on exogenous BMP4 stimulation [4], which, although necessary, is not sufficient to fully provide the extent of signals required for robust PGC-LC induction, survival, and proliferation. An emerging strategy has been the use of conditioned medium from somatic or stem cell sources, such as cumulus cells [5] or testicular cells [6]. Recent in vitro models have attempted to recapitulate the amniotic niche by first differentiating human embryonic stem cells toward amniotic epithelium–like cells and subsequently inducing primordial germ cell-like cells through paracrine signaling within the same culture system [7]. While this approach provides valuable insight into early germline specification, it relies on complex three-dimensional aggregate cultures that introduce additional technical variability.

As mentioned above, primate PGC specification takes place in the dorsal amnion. Based on this knowledge, we report for the first time the use of human amniotic fluid stem cell (hAFSC) conditioned medium to mimic the in vivo niche signaling of PGCs in primates. hAFSCs represent a unique source of paracrine factors, some of which are analogous to those present in the embryonic germ cell niche. The hAFSC secretome is rich in cytokines, growth factors, extracellular vesicles, and microRNAs [8]. Proteomic and transcriptomic profiling of hAFSC-derived secretome has demonstrated secretion of stem cell factor (SCF), leukemia inhibitory factor (LIF), basic fibroblast growth factor (bFGF), vascular endothelial growth factor (VEGF), hepatocyte growth factor (HGF), WNT ligands, and TGF-β family members, many of which have been implicated in germ cell specification and survival [9,10,11]. In 2025, Phermthai et al. published a detailed proteomic analysis of the hAFSC secretome, demonstrating that it contains more than 2000 distinct proteins [12]. Moreover, extracellular vesicles released by hAFSCs contain microRNAs and RNA-binding proteins that can potentially be involved in the modulation of germline-related signaling pathways.

This secretory profile parallels the paracrine support provided by extraembryonic tissues during early embryogenesis, thereby offering a physiologically relevant alternative for in vivo germline niche conditions. In particular, SCF-KIT and LIF-STAT3 signaling axes, both abundant in the hAFSC secretome, are known to support PGC proliferation and maintenance [9], while WNT and BMP pathways jointly regulate germline induction [13].

hAFSCs, however, are intrinsically heterogeneous, consisting of mixed populations with mesenchymal-, epithelial-, and endothelial-like characteristics that vary in their differentiation potential and secretory output [14]. This heterogeneity can complicate experimental reproducibility, as the composition of the conditioned medium depends on the relative abundance of these subpopulations. Sorting hAFSCs for the SSEA4-positive fraction provides a strategy to reduce this variability [15]. Following SSEA-4 sorting, the cell culture becomes enriched in cells exhibiting more uniform basal states, minimizing variability introduced by minor subpopulations. Consequently, the conditioned medium produced displays a more consistent profile of growth factors, exosomes, cytokines, and metabolites, greatly enhancing experimental reproducibility and establishing a more stable and reliable baseline secretome.

In this protocol, we combine BMP4 stimulation with hAFSC-4 conditioned medium to induce differentiation of hESCs into DDX4/VASA- and DAZL-positive PGC-LCs using adherent cell cultures. While BMP4 provides the essential trigger for germline specification, the hAFSC-4 secretome contributes additional supportive signals that enhance cell survival and promote a niche-like environment, thereby improving the efficiency and fidelity of in vitro PGC-LC generation. This strategy more closely mimics the synergistic signaling environment of the embryonic niche, offering a robust model for dissecting the molecular mechanisms of human germline development.

Another key aspect of our protocol that ensures reproducibility is the use of adherent cultures instead of embryoid body (EB) formation. EB formation is intrinsically spontaneous and stochastic, resulting in variability in size, cell composition, and microenvironmental signaling, which can undermine reproducibility. In contrast, adherent cultures provide a more uniform environment, promoting consistent cell–cell and cell–matrix interactions, controlled exposure to differentiation factors, and a predictable microenvironment. This strategy markedly improves reproducibility and enables the reliable generation of PGC-LCs. The reported reproducibility refers to consistency across independent experiments within the B1 hESC line rather than cross-line validation.

## 2. Experimental Design

**Figure 1:**
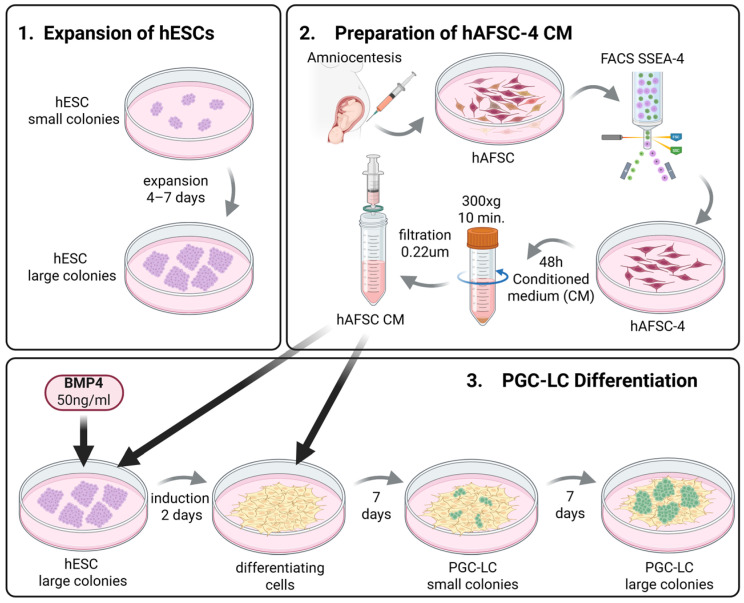
Schematic overview of the protocol. Two preparatory phases (1 and 2) precede phase 3, which details the steps culminating in PGS-LC formation. Black arrows indicate the addition of factors that trigger differentiation. Small gray arrows indicate cell culture progression steps and time intervals. Cell states are color-coded: undifferentiated stem cells in purple, differentiating ES cells in yellow, and PGS-LC in green.

The workflow comprises three main phases (Figure 1).

1. Expansion of hESCs: hESC line B1 is expanded under feeder-free conditions on Matrigel in mTeSR1 medium.
2. Preparation of hAFSC-4 CM: hAFSCs are isolated from first-trimester amniotic fluid, expanded, and SSEA4-positive cells are enriched by flow cytometry. Conditioned medium is collected after 48 h of culture.
3. PGC-LC Differentiation: hESCs are induced with BMP4 (50 ng/mL) in hAFSC-4 CM, followed by continued culture in hAFSC-4 CM alone for 14 days. Cells are harvested on day 16 for immunofluorescence staining of DDX4/VASA and DAZL. Positive controls include murine postnatal testicular germ cells.
4. Expected outcome: Small groups of PGC-LCs can be observed as early as day 9, comprising round, phase-bright cells. By day 16, these groups expand into large clusters of PGC-LCs, positive for DDX4/VASA and DAZL.

### 2.1. Materials

Cells and cell culture media:

1. Human embryonic stem cell line B1. The human embryonic stem cell line B1 was derived and characterized in our laboratory as part of a previous project, Grant Б02-13/2014, supported by the Bulgarian National Science Fund, and it was used consistently in all experiments.
2. Human amniotic fluid stem cells (hAFSCs) were derived from two donors with a gestational age of 15 weeks. The hAFSCs used in the study were obtained with informed consent as residual biological material after prenatal genetic diagnosis performed at the Specialized Hospital for Active Treatment in Obstetrics and Gynecology “Maichin Dom”.
3. mTeSR1 medium (StemCell Technologies, Vancouver, BC, Canada; Cat.# 85850).
4. Minimum Essential Medium α (α-MEM) (Gibco, Inchinnan, Scotland, UK; Cat.# 12571063).
5. Dulbecco’s Modified Eagle Medium (DMEM), high glucose (Gibco, Inchinnan, Scotland, UK; Cat.# 11965092).
6. CHANG medium B (Irvine Scientific, Santa Ana, CA, USA; Cat.# C100).
7. CHANG medium C (Irvine Scientific, Santa Ana, CA, USA; Cat.# C106).
8. Complete hAFSC medium (α-MEM + 10% FBS + 50 U/mL Pen/Strep + 18% Chang B + 2% Chang C).

Plasticware:

1. Nunc 4-well plates (Nunc, Roskilde, Denmark; Cat.# 176740).
2. Corning 6-well plates (Corning, Reynosa, Tamaulipas, Mexico; Cat.# 3516).
3. Glass bottom 24-well plates (Greiner, Frickenhausen, Germany; Cat.# 662892).

Antibodies:

1. Anti-DDX4/MVH (Abcam, Cambridge, UK; Cat.# ab13840).
2. Anti-DAZL (Abcam, Cambridge, UK; Cat.# ab34139).
3. Anti-SSEA4 antibody [MC813-70] (Abcam, Cambridge, UK; Cat.# ab16287).
4. Cy™3 AffiniPure^®^ Goat Anti-Mouse IgG (H + L) (Jacksonimmuno, West Grove, PA, USA; Cat.# 115-165-003).
5. FITC anti-human SSEA-4 Antibody (BioLegend, San Diego, CA, USA; Cat.# 330410).
6. Cy™2 IgG Fraction Monoclonal Mouse Anti-Rabbit (Jacksonimmuno, West Grove, PA, USA; Cat.# 211-222-171).

Other reagents:

1. Fetal bovine serum (HyClone, Logan, UT, USA; Cat.# SH3007103).
2. ReLeSR (StemCell Technologies, Vancouver, BC, Canada; Cat.# 100-0483).
3. Matrigel (Corning, Bedford, MA, USA; Cat.# 354277).
4. Penicillin–Streptomycin (Gibco, Grand Island, NY, USA; Cat.# 15140122).
5. Recombinant human BMP4 (R&D systems, Minneapolis, MN, USA; Cat.# 314-BP).
6. PBS, Ca^2+^/Mg^2+^-free (Gibco, Inchinnan, Scotland, UK; Cat.# 14-190-144).
7. Trypsin–EDTA (Gibco, Inchinnan, Scotland, UK; Cat.# 15400054).
8. Collagenase, Type IV, powder (Gibco, Grand Island, NY, USA; Cat.# 17104019).
9. Bovine serum albumin (Sigma-Aldrich, St. Louis, MO, USA; Cat.# 126615-25ML).
10. 0.22 µm filters (Thermofisher, Suzhou, China; Cat.# 723-9920).
11. 4% paraformaldehyde (Sigma-Aldrich, St. Louis, MO, USA; Cat.# 1004968350).
12. Triton X-100 (Sigma-Aldrich, St. Louis, MO, USA; Cat.# T8787-100ML).
13. DAPI nuclear stain (Invitrogen, Eugene, OR, USA; Cat.# D1306).

### 2.2. Equipment

1. Biosafety cabinet (BioAir, model: AURA VIP 1.8 BASIC LED, made in Italy),
2. CO_2_ incubator, 37 °C, 5% CO_2_, humidified (PHCBI, model: MCO-50AIC-PE, designed in Japan, made in Indonesia)
3. Flow cytometer with sorting capability (SONY, model: LE-SH800SEP, made in Japan)
4. Fluorescence microscope (GE Healthcare, model: DeltaVision Ultra, WA 98027 USA)
5. Centrifuge (OHAUS, model: FC5718R, made in Germany)
6. Serological Pipettes (Nunc, Roskilde, Denmark) and sterile consumables

## 3. Procedure

### 3.1. Maintenance of hESCs (Line B1) on Matrigel with mTeSR1 Medium

1. Thaw and expand hESCs on Matrigel-coated 4-well plates in mTeSR1 (500 µL per well) at 37 °C and 5% CO_2_, following the manufacturer’s instructions. Feed cells daily with fresh mTeSR1 medium.
2. Passage colonies every 4–6 days using ReLeSR, following the manufacturer’s instructions, when they reach ~70–80% confluence.
3. To prepare hESCs for PGC-LC induction, transfer them in Matrigel-coated glass-bottom 24-well plates in mTeSR1 medium. Feed cells daily with fresh mTeSR1 medium until they reach ~70% confluence.

### 3.2. Derivation and Expansion of hAFSCs

Adopted from De Coppi P. et al. (2007) [16] with modifications. Collect amniotic fluid (AF) by standard amniocentesis.

**Figure 2:**
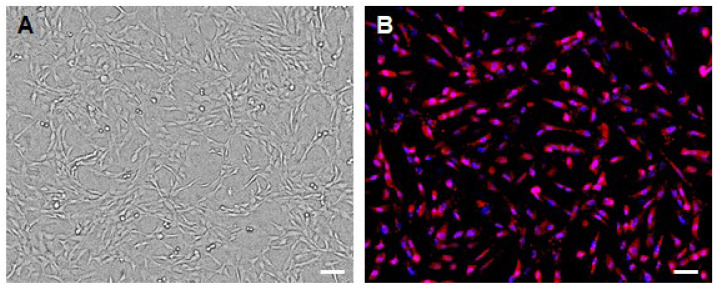
Human amniotic fluid stem cells are used for the production of conditioned medium. (**A**) Phase-contrast image of hAFSCs demonstrating their typical mesenchymal morphology. (**B**) Immunofluorescence image obtained using anti–SSEA-4 antibodies (red) and DAPI (blue) to visualize cell nuclei. After sorting, all cells in the resulting population express SSEA-4, although with varying intensity. Scale bar: 100 μm.

1. Centrifuge AF at 300× *g* for 10 min, discard supernatant, and resuspend the cell pellet in complete hAFSC medium (α-MEM + 10% FBS + 50 U/mL Pen/Strep + 18% Chang B + 2% Chang C).
2. Plate cells in 6-well plates (3 mL per well) at 1.5 × 10^4^ cells per cm^2^ and incubate at 37 °C, 5% CO_2_. Replace medium every 2 days.
3. When cultures reach ~70% confluence, wash the cells with PBS pre-warmed to 37 °C.
4. Incubating them for 3 min. in 0.05% (1×) trypsin-EDTA at 37 °C.
5. Add DMEM + 10 FBS% to stop the process and centrifuge the cells at 250× *g* for 10 min.
6. Discard the supernatant and resuspend the cells in complete hAFSC medium. Plate cells in 6-well plates (3 mL per well) at 1.5 × 10^4^ cells per cm^2^ and incubate at 37 °C, 5% CO_2_. Replace medium every 2 days.
7. Expand the cultures through three passages to enrich proliferative hAFSCs. These cells exhibit mesenchymal morphology (Figure 2A).

### 3.3. FACS Sorting of SSEA-4 Positive hAFSCs (hAFSC-4)

1. Harvest passage-3 hAFSCs with trypsin-EDTA, and wash cells twice in PBS with 1% BSA.
2. Incubate the single-cell suspension with FITC anti-human SSEA-4 Antibody (BioLegend, San Diego, CA, USA; Cat.# 330410) (5 µL per million cells in 100 µL staining volume) for 30 min at 37 °C in PBS containing 2% FBS (protected from light).
3. Wash twice in PBS with 1% BSA to remove unbound antibody.
4. Sort cells on a flow cytometer, gating to collect the SSEA-4 positive fraction (hAFSC-4) (FACS gating: Include both unstained and isotype controls for SSEA-4 to properly define and set sorting gates.) Collect hAFSC-4 cells into complete hAFSC medium and plate them in 6-well plates (3 mL per well) at 1.5 × 10^4^ cells per cm^2^.
5. To confirm successful sorting, perform immunofluorescence staining using an anti-SSEA4 antibody [MC813-70] (Abcam; Cat.# ab16287) and a Cy™3 AffiniPure^®^ Goat Anti-Mouse IgG (H + L) (Jacksonimmuno; Cat.# 115-165-003) secondary antibody (Figure 2B). For details, see Section 3.7.

### 3.4. Preparation of Conditioned Medium (hAFSC-4 CM)

For consistency, passage numbering should start after SSEA-4 sorting, and hAFSC-4 cells between passages 2 and 4 should be used for the preparation of conditioned medium.

**Figure 3:**
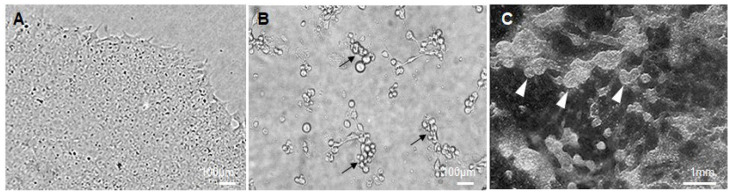
Change in cell morphology during hESCs differentiation to PGC-LC (**A**) Typical view of hESCs colony before induction of differentiation. (**B**) Cell morphology of hESCs on day 9 after induction with BMP4 for 48 h and subsequent cultivation in hAFSC-4 CM for 7 days, characterized by the formation of groups of rounded, phase-bright differentiating cells (black arrows). (**C**) By day 16, the cells form clearly distinguishable large clusters of PGC-LCs protruding above the basal cell layer (white arrowheads).

1. When hAFSC-4 cultures reach ~70% confluence in a 6-well plate, aspirate old medium and add 3 mL of fresh complete hAFSC medium.
2. Incubate for 48 h. Collect the conditioned medium and centrifuge at 300× *g* for 10 min to remove any cells and debris.
3. Filter the supernatant through a 0.22 µm filter to ensure that no hAFSCs remain in the conditioned medium. This filtered medium is the hAFSC-4-conditioned medium (hAFSC-CM), rich in hAFSC-secreted factors. (Functional bioactivity assessment of conditioned medium: To verify the bioactivity of each batch of hAFSC-4 conditioned medium (hAFSC-4 CM) prior to full-scale differentiation, a small-scale pilot assay (4-well plate) is recommended. Briefly, hESCs are exposed to hAFSC-4 CM supplemented with BMP4 (50 ng/mL) under standard differentiation conditions for 48 h and then cultured in hAFSC-4 CM alone for an additional 6–8 days. Cell survival and morphology are monitored throughout this period. Bioactive hAFSC-4 CM consistently supports early cell viability and promotes the appearance of characteristic, rounded, phase-bright cell clusters within the first 7–9 days of differentiation (Figure 3B). Failure to observe these features may indicate reduced hAFSC-4 CM activity and warrants the preparation of a new hAFSC-4 CM batch before proceeding with downstream experiments.)
4. Use hAFSC-4 CM immediately or store at 4 °C short-term (≤24 h) or freeze aliquots at −20 °C for longer storage.

### 3.5. Induction of PGC-LCs Differentiation from hESCs

1. On Day 0, ensure that hESC cultures growing in glass-bottom 24-well plates consist of undifferentiated colonies measuring 1–1.5 mm in diameter and not contacting each other (Figure 3A). (Cell density and colony quality of hESCs: Optimal results may depend on the initial hESC plating density and colony quality. Cultures that are too sparse or too confluent, as well as poor-quality hESC colonies, can negatively affect PGC-LCs differentiation efficiency.)
2. At this point, aspirate the mTeSR1 medium from the glass-bottom 24-well plate and gently wash the cells once with PBS pre-warmed to 37 °C.
3. Add hAFSC-4 CM (500 µL per well) supplemented with recombinant human BMP4 at 50 ng/mL (BMP4 handling: Reconstitute BMP4 in 0.1% BSA in PBS to stabilize the protein, and prepare single-use aliquots to avoid repeated freeze–thaw cycles), and return the cultures to the incubator.
4. After 48 h (Day 2), carefully replace the medium with fresh hAFSC-4 CM without BMP4. Continue culturing the cells in hAFSC-4 CM alone, replacing the medium every 2 days with fresh hAFSC-4 CM.
5. By Day 9, small groups of PGC-LCs comprising round, phase-bright cells can be observed (Figure 3B). Maintain the differentiation culture until Day 16, when PGC-LCs proliferate to form large clusters that protrude above the basal cell layer (Figure 3C).

### 3.6. Preparation of Mouse Germ Cells

1. Mouse testes are collected under sterile conditions and decapsulated.
2. Seminiferous tubules are enzymatically dissociated using Collagenase IV 100 U/mL for 30 min, followed by Trypsin–EDTA for 7 min at 37 °C to obtain a single-cell suspension.
3. Cells are filtered through a 100 µm cell strainer.
4. The suspension is centrifuged at 300× *g* for 10 min.
5. Cells are plated in DMEM + 10% FBS + 50 U/mL Pen/Strep.
6. Cultures are maintained at 37 °C with 5% CO_2_.
7. The culture medium is changed every second day.

### 3.7. Immunofluorescence Analysis of DDX4/VASA, DAZL and SSEA-4

Since the steps for immunofluorescence analysis are common to all three cell types analyzed, each step describes the shared procedure and additionally highlights any differences specific to each cell type.

1. Gently wash the cell cultures (e.g., PGC-LCs, hAFSCs, or mouse germ cells) with PBS, then fix with 4% paraformaldehyde in PBS for 12 min at room temperature.
2. Wash twice with PBS, then permeabilize with 0.1% Triton X-100 in PBS for 10 min at room temperature (permeabilization is not required for SSEA-4 staining).
3. Block non-specific binding in PBS containing 1% BSA for 30 min at room temperature.
4. For detection of DDX4/VASA, incubate samples (PGC-LCs or mouse germ cells) with the primary anti-DDX4/MVH antibody (Abcam, Cat. #ab13840; 1:200 dilution) diluted in blocking solution, overnight at 4 °C.
5. For detection of DAZL, incubate samples (PGC-LCs or mouse germ cells) with the primary anti-DAZL antibody (Abcam, Cat. #ab34139; 1:200 dilution) diluted in blocking solution, overnight at 4 °C.
6. For detection of SSEA-4, incubate samples (hAFSC) with the primary anti–SSEA-4 antibody [MC813-70] (Abcam, Cat. #ab16287; 1:200 dilution) diluted in blocking solution, overnight at 4 °C.
7. After incubation with the appropriate primary antibody, wash samples three times with PBS and incubate with the appropriate fluorescent secondary antibodies (Cy™2 IgG Fraction Monoclonal Mouse Anti-Rabbit (Jacksonimmuno; Cat.# 211-222-171) for DDX4/VASA and DAZL or Cy™3 AffiniPure^®^ Goat Anti-Mouse IgG (H + L) (Jacksonimmuno; Cat.# 115-165-003) for SSEA-4) for 1 h at room temperature in the dark.
8. Wash three times with PBS and counterstain nuclei with 0.5 µg/mL DAPI for 10 min in the dark.
9. Mount coverslips with antifade reagent and image using fluorescence microscopy.

**Figure 4:**
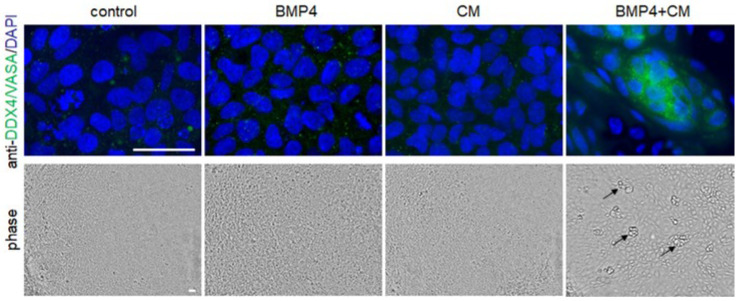
Effect of bone morphogenetic protein 4 (BMP4), hAFSC-4 conditioned medium (CM), and their combination (BMP4 + CM) on DDX4/VASA (green) expression nine days after treatment initiation (day 9). Cell nuclei were counterstained with DAPI (blue). The bottom row shows corresponding phase-contrast images illustrating treatment-induced morphological changes (black arrows). Notably, DDX4/VASA expression was observed only in cells subjected to the combined BMP4 + CM treatment. Scale bar: 50 µm.

Note that the primary antibodies used for anti-DDX4/VASA and anti-DAZL are validated for cross-reactivity in both human and murine samples (Mouse germ cells as a positive control: Use primary cultures of mouse postnatal testicular germ cells stained in parallel as positive controls for antibody validation), as supported by manufacturer documentation. Negative controls included treatment with complete hAFSC medium (control), complete hAFSC medium supplemented with 50 ng/mL BMP4 (BMP4), hAFSC-4 conditioned medium (CM), and the combination of BMP4 and hAFSC-4 conditioned medium (BMP4 + CM). Notably, only the combined BMP4 + CM treatment was sufficient to support PGC-LS differentiation and survival (Figure 4).

## 4. Expected Result

**Figure 5:**
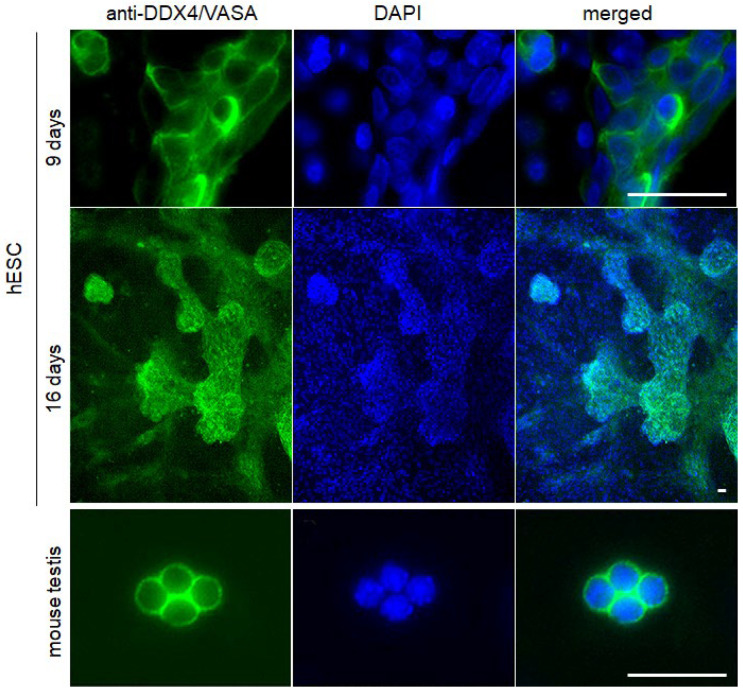
Expression of DDX4/VASA (anti-DDX4/VASA) in the cytoplasm of cells differentiated from hESCs is detectable at the ninth day (9 days) of differentiation, and large clusters of PGC-LC become well-developed after an additional seven days (16 days). Primary cell cultures from the mouse testis at day 4 were used as a positive control (mouse testis). Cell nuclei were visualized by DNA staining with DAPI. Scale bar: 50 μm.

**Figure 6:**
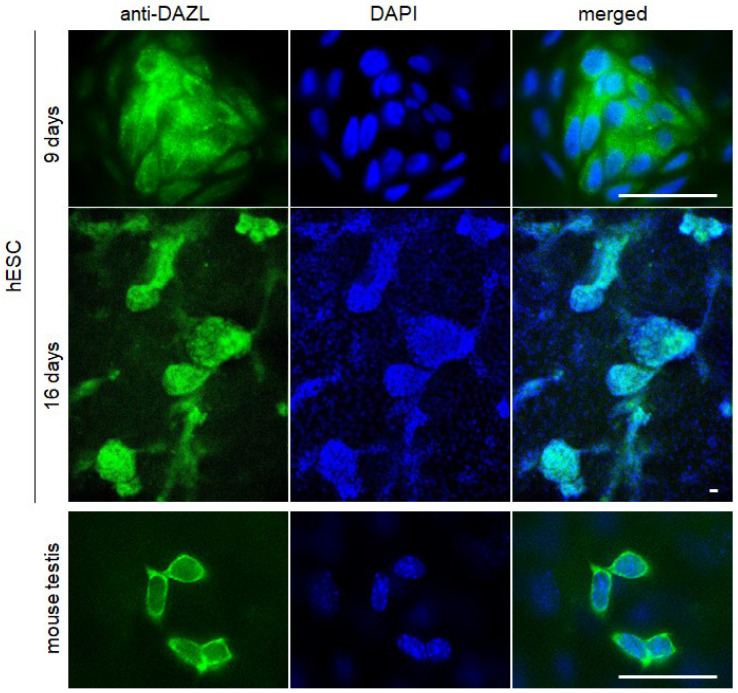
Cytoplasmic DAZL expression (anti-DAZL) in treated human embryonic stem cells (hESCs) becomes detectable by day 9 after the onset of differentiation, and prominent clusters of PGC-like cells (PGC-LCs) are well developed after an additional seven days (day 16). Primary cultures of mouse testis cells at day 4 served as a positive control. Cell nuclei were counterstained with DAPI. Scale bar: 50 μm.

At Day 16, between 40 and 60% of cells will exhibit cytoplasmic expression of DDX4/VASA and DAZL. In summary, BMP4 + hAFSC-4 CM treatment should yield clearly distinguishable large clusters of PGC-LCs protruding above the basal cell layer, consisting of DDX4/VASA (Figure 5) and DAZL (Figure 6) positive PGC-LCs by Day 16.

## 5. Conclusions

This study establishes an in vitro protocol for generating PGC-PCs from hESCs using BMP4 in combination with conditioned medium derived from SSEA-4–enriched hAFSCs. The differentiation is based on adherent cultures and avoids embryoid body formation, thereby reducing variability associated with three-dimensional aggregation [17] and improving experimental consistency.

Under these conditions, hESCs gave rise to morphologically distinct cell clusters and exhibited cytoplasmic expression of the germline-associated markers DDX4/VASA and DAZL by day 16 of differentiation. The use of hAFSC-conditioned medium likely provides additional paracrine support that complements BMP4-mediated induction and promotes PGC-LC differentiation, survival, and proliferation. Since all experiments were performed using a single hESC line, additional validation across additional hESC and iPSC lines represents an important future extension of the protocol.

The described protocol represents a technically accessible and reproducible framework for human germ cell–like differentiation. As such, it can serve as a practical platform for subsequent molecular, transcriptional, epigenetic, and functional analyses aimed at further characterizing germline specification and maturation in vitro.

### Notes

Note 1: Cell density and colony quality of hESCs: Optimal results may depend on the initial hESC plating density and colony quality. Cultures that are too sparse or too confluent, as well as poor-quality hESC colonies, can negatively affect PGC-LCs differentiation efficiency.

Note 2: BMP4 handling: Reconstitute BMP4 in 0.1% BSA in PBS to stabilize the protein, and prepare single-use aliquots to avoid repeated freeze–thaw cycles.

Note 3: Mouse germ cells as a positive control: Use primary cultures of mouse postnatal testicular germ cells stained in parallel as positive controls for antibody validation

Note 4: Functional bioactivity assessment of conditioned medium: To verify the bioactivity of each batch of hAFSC-4 conditioned medium (hAFSC-4 CM) prior to full-scale differentiation, a small-scale pilot assay (4-well plate) is recommended. Briefly, hESCs are exposed to hAFSC-4 CM supplemented with BMP4 (50 ng/mL) under standard differentiation conditions for 48 h and then cultured in hAFSC-4 CM alone for an additional 6–8 days. Cell survival and morphology are monitored throughout this period. Bioactive hAFSC-4 CM consistently supports early cell viability and promotes the appearance of characteristic rounded, phase-bright cell clusters within the first 7–9 days of differentiation (Figure 3B). Failure to observe these features may indicate reduced hAFSC-4 CM activity and warrants the preparation of a new hAFSC-4 CM batch before proceeding with downstream experiments.

Note 5: FACS gating: Include both unstained and isotype controls for SSEA-4 to properly define and set sorting gates.

## Data Availability

The raw data supporting the conclusions of this article will be made available by the authors on request.
